# Construction of nomogram for wound recurrence in elderly patients with venous leg ulcers

**DOI:** 10.3389/fmed.2024.1401280

**Published:** 2024-10-30

**Authors:** Wenfang Mu, Anzi Wang, Zhiwei Xu, Yan Wang, Li Xu, Xian Wang

**Affiliations:** ^1^Department of Nursing, Renji Hospital, School of Medicine, Shanghai Jiao Tong University, Shanghai, China; ^2^School of Nursing, Gansu University of Chinese Medicine, Lanzhou, China; ^3^School of Nursing, Shanghai University of Traditional Chinese Medicine, Shanghai, China; ^4^Department of Nursing, Shanghai Municipal Hospital of Traditional Chinese Medicine, Shanghai, China

**Keywords:** elderly patients, venous leg ulcers, VLUs, Wound recurrence, prediction model, nomogram

## Abstract

**Background:**

Venous Leg Ulcers (VLUs) are one of the most serious and intractable complications of chronic venous insufficiency. This study aims to develop a nomogram based on a theoretical model to predict the probability of wound recurrence in older patients with VLUs.

**Methods:**

The elderly patients with VLUs attending the five hospitals between September 2021 and October 2022 were enrolled in this research, and randomized to the training and validation cohorts based on the corresponding ratio (7:3). Recurrent events were recorded during a six-month follow-up after the baseline data collection. The univariate analysis, the least absolute shrinkage and selection operator (LASSO) regression method were used to screen variables, and multiple logistic regression was used to establish a risk prediction model, which was presented by nomogram. Receiver operating curves (ROC), Hosmer–Lemeshow test, as well as calibration curves, were adopted to assess the effectiveness of the nomogram. The prognostic value of the nomogram was also examined.

**Results:**

A total of 608 elderly patients with VLUs were included in the study. They were randomly divided into the training cohort (*N* = 421) and the validation cohort (*N* = 187). In the training cohort, Lasso regression and multivariate logistic regression analysis indicated that previous recurrence number, last ulcer duration, lower extremity DVT history, and frailty were independent risk factors for wound recurrence in elderly patients with VLUs, while daily exercise time and self-efficacy were protective factors. A nomogram was established with a good discrimination capacity and predictive efficiency with and the area under the curve (AUC) of 0.869 (95%CI: 0.831–0.908) in the training set and 0.890 (95%CI: 0.841–0.938) in the validation set. The *p* values of the Hosmer-Lemeshow test for both sets were 0.887 and 0.772, respectively, both greater than 0.05. The calibration degree charts showed that the data point connection was similar to the diagonal, indicating that the model’s prediction probability of wound recurrence in elderly VLUs patients is close to the actual probability.

**Conclusion:**

This study constructed a new nomogram to predict the risk of wound recurrence in elderly patients with VLUs. The nomogram has excellent accuracy and reliability, which can help healthcare workers and patients actively monitor and follow up with patients to prevent the recurrence of ulcers and make clinical decisions.

## Introduction

Venous Leg Ulcers (VLUs) pose significant and challenging complications associated with chronic venous insufficiency, manifesting as open skin lesions on the legs or feet due to venous hypertension (1). The global prevalence of VLUs is estimated to be between 1 and 3% (2), constituting approximately 80% of all leg ulcers (1). The prevalence of VLUs tends to rise with age, reaching 3 to 4% in individuals aged 65 and older (3, 4). VLUs are characterized by slow healing and a high recurrence rate. Research indicates that 75% of patients experience a relapse within 3 weeks after VLU healing (5), and the recurrence rate within 6 months ranges between 50 and 70% (1). This recurrence rate is even higher in elderly patients. Over a 10-year period, 10% of patients endure recurring VLUs, severely impacting their quality of life (6). Additionally, 28% of patients experience more than 10 recurrences of ulcers throughout their lifetime (7).

Symptoms such as pain, itching, and frequent recurrence after treatment significantly impact patients’ quality of life and mental health (8). For patients with VLUs, the long-term presence and frequent recurrence of ulcers can result in feelings of uncertainty, disappointment, and hopelessness. These patients often fear that they will never be able to overcome this situation (9, 10). Moreover, the frequent recurrence of VLUs places a significant financial burden on patients’ families and society due to the high cost of treatment and nursing (11).

Several studies have confirmed that various factors contribute to the recurrence of ulcers, including ulcer healing time, lack of surgical treatment, BMI, history of deep vein thrombosis (DVT), ulcer size, lack of compression therapy, calf muscle exercise, and social support (12, 13). However, surgical treatment may not be suitable for elderly patients due to their age and concomitant diseases. Moreover, it has been found that addressing adverse behavioral factors can effectively reduce the risk of recurrence (14). Despite extensive research, identifying the most reliable biomarkers for wound recurrence remains challenging. It is important to note that relying on a single risk factor alone does not provide an accurate prediction of recurrence risk in elderly VLUs patients. Therefore, a comprehensive and systematic exploration of the various factors influencing wound recurrence within a theoretical framework is crucial. Such an approach will help us better understand the complexity of the VLUs recurrence process and enable the implementation of relevant interventions based on identified risk or protective factors.

The tool for predicting the risk of wound recurrence in VLUs patients was only studied by a team in Australia (15). However, the characteristics of elderly patients were not given attention, and no relevant risk prediction studies were found in China. Therefore, this study aimed to construct a risk prediction model for wound recurrence in elderly VLUs patients under the guidance of the health ecology model. The objective was to provide a scientific basis for medical staff to assess the risk of wound recurrence in elderly VLUs patients and offer evidence support for targeted wound recurrence intervention programs.

## Methods

### Study population

The study surveyed elderly patients with VLUs who visited five hospitals in Shanghai between March 2022 and October 2022. The researchers provided an introduction to the study and obtained informed consent from the patients, who then completed the questionnaire either independently or with the assistance of the researchers. The study received approval from the Medical Ethical Committee of Shanghai Hospital (2022SHL-KY-51-01). The inclusion criteria for this study were as follows: (1) The wound had healed, defined as achieving 100% epithelialization and being maintained for at least 2 weeks; (2) Age ≥ 60 years; (3) Ankle-brachial index >0.9; (4) The patient voluntarily participated in the study and provided informed consent. The exclusion criteria included: (1) Patients with vital organ failure, such as severe heart, liver, lung, or renal damage; (2) Wounds associated with carcinogenesis.

### Selection of predictor variables

A systematic review of the literature was conducted in January 2022 to identify recurrence risk factors that have substantial evidence supporting their inclusion as predictor variables in the risk model. Subsequently, the influencing factors obtained from the literature review were integrated with the theoretical framework of health ecology and clinical characteristics to establish the predictive variables for this study.

### Data collection

The baseline data were collected including age, gender, BMI, education, marital status, place of residence, living status, occupation, average monthly family income, payment method, number of years with VLUs, number of previous recurrences, duration of the last ulcer, largest area of the last ulcer, venous surgery, history of DVT in the lower extremity, heart disease, rheumatoid arthritis, diabetes, hypertension, anemia, cancer, renal dysfunction, smoking status, alcohol consumption status, daily leg elevation time, daily exercise time, daily stress treatment time, daily living ability, nutrition, frailty, social support, depression, and self-efficacy. Given that the focus of this study is on the elderly, who typically have limited reserves, it is essential to keep the data collection time brief. Consequently, we collected data through face-to-face interviews with patients regarding their daily leg elevation time, daily exercise time, daily stress treatment time, daily living abilities, nutritional status, frailty, social support, depression levels, and self-efficacy. Additionally, demographic and disease information, such as gender, age, and the duration of the last ulcer, was extracted from the hospital information system. On average, the time spent on data collection through face-to-face interviews in this study was 19.2 ± 7.6 min, excluding the time spent downloading data from hospital information systems.

Daily Living Ability: Activities of Daily Living Scale (ADL) was adopted (16). ADL consists of the Physical Self Maintenance Scale (PSMS) and Instrumental Activities of Daily Living Scale (IADL), a total of 14 items. Each item is rated 1–4 points according to the Likert 4-level scoring method, which is completely capable of doing it by itself, somewhat difficult, needing help, and unable to do it at all. The total score of this scale is 14 ~ 56 points, > 14 points have different degrees of dysfunction, and ≥ 22 points are obvious dysfunction.

Nutrition: The Mini-Nutritional Assessment Short-Form (MNA-SF) was used. The scale was formed by Rubenstein et al. (17) by simplifying the traditional MNA, including six aspects of food intake and food intake reduction in the past 3 months, weight loss, activity ability, acute illness or psychological trauma, mental and psychological problems, and BMI. A 4-level scoring method was adopted (0–3 points), with a total score of 0–14 points. Among them, 0 to 7 were classified as undernourished, 8 to 11 as at risk of malnutrition, and 12 to 14 as normal nutritional status.

Frailty: This study used the Frail scale (18) to evaluate frailty. This scale was jointly proposed by members of six authoritative geriatric expert groups and has been recommended as an effective tool for early screening for the debility of elderly people in the community. The scale is composed of 5 items, “yes” is 1 point, “no” is 0 points, the total score is 5 points, ≥3 points exist frailty, ≤2 points do not exist frailty, the higher the score, the higher the probability of fragility.

Depression: The Geriatric Depression Scale-15 (GDS-15) was used. GDS-15 was simplified and formed on the GDS scale made by Sheikh et al. (19) and Brink et al. (20). It is suitable for the elderly to assess their depression in the past week and can cover the core manifestations of depression in the elderly. The scale includes 15 items, and the total score is 0 to 15, with ≥8 indicating the presence of depressive symptoms.

Self-efficacy: Use the General Self-efficacy Scale (GSES). There are 10 items in this scale, and the score is Likert 4-level scoring method (1–4 points). All options are scored in reverse, and the total score is 10–40 points. The total mean score method is the sum of the scores of all items divided by 10, the total mean score is high, and the sense of self-efficacy is high. The total average score of 1 ~ 2 is classified as low level, 2.1 ~ 3 is classified as medium level, and 3.1 ~ 4 is classified as high level.

Social Support: The Social Support Rating Scale (SSRS) was adopted. It includes 3 dimensions of subjective support, objective support, and social support utilization, a total of 10 items. The total score ranges from 12 to 66, with a higher score indicating a higher level of social support. Low, medium, and high levels of social support correspond to a total score of 22 or less, 23 to 44, and 45 to 66, respectively.

### Clinical outcomes

The primary study endpoint was recurrence, which was defined as the reappearance of a wound of venous etiology on the same leg that was previously affected by VLUs. The researchers conducted telephone interviews and outpatient follow-ups at 3 and 6 months after the baseline investigation. The follow-up visits were scheduled within a deviation of no more than 7 days. If the subjects’ wounds recurred, they were no longer followed up, indicating that they had reached their natural endpoint.

Standardized training manuals, operation manuals, questionnaires, and form-filling instructions were utilized for both baseline investigation and follow-up. Prior to the investigation, all participants underwent specialized training for investigators, and were required to pass an examination to qualify for participation. During the on-site investigation, the team arranged for supervisors to conduct irregular tour supervision and randomly selected survey data to verify the accuracy and completeness of the information. They also performed logical tests on the data and reported any issues on the spot. To assess the reliability of the measurements, the team sampled 5% of the questionnaires completed on the same day and conducted independent repeated measurements on the selected subjects to compare the consistency of the two measurement results. All data were double-entered and subjected to logical checks by professional data personnel, who promptly identified and corrected any errors.

### Statistical analysis

All data were analyzed using SPSS23.0, R4.2.2, and R studio software. Count data were statistically described using frequency and composition ratios. The Chi-square test was used to compare counting data between two groups. The data set was randomly divided into training and validation sets in a 7:3 ratio, in which the training set was used to construct the prediction model and the validation set was used to verify and evaluate the model performance. Variable screening was performed through single-factor analysis to identify statistically significant variables (*p* < 0.05). From the meaningful variables in the single-factor analysis, candidate variables were selected and combined with professional knowledge. The Lasso algorithm was then used to determine the optimal number of variables to include in the final model, to avoid overfitting. The risk prediction model was established using binary Logistic multivariate regression analysis. Finally, we quantified the model’s ability to distinguish between classes using the Area Under the Receiver Operating Characteristic (ROC) Curve (AUC). The goodness-of-fit of the model was assessed by the Hosmer-Lemeshow test, with a *p*-value >0.05 indicating an acceptable model fit. Additionally, a calibration curve was plotted to assess the calibration ability of the models. Then, the model was presented as a Nomogram.

## Results

### Baseline clinical characteristics

In the baseline data collection stage, incomplete or inconsistent questionnaire responses were excluded, and complete baseline data were collected from 636 cases. Out of the initial 636 cases, 28 patients were lost to follow-up, resulting in 608 elderly VLUs patients included in the study. Among these patients, 443 (72.9%) experienced recurrence. The 608 patients were randomly divided into training set (*n* = 421) and verification set (*n* = 187) according to the ratio of 7:3. Baseline Clinical Characteristics of elderly VLUs patients in the training and validation sets are shown in Table 1, and most of the variables included were evenly distributed.

**Table 1:** Baseline characteristics of patients in the training set, validation set, and all populations.

	Characteristic	Total (*n* = 608)	Training set (*n* = 421)	Verification set (*n* = 187)	*p-*value
Gender	Male	376 (61.8)	263 (62.5)	113 (60.4)	0.632
	Female	232 (38.2)	158 (37.5)	74 (39.6)	
Age	60–69	250 (44.1)	175 (41.6)	75 (40.1)	0.866
	70–79	202 (33.2)	137 (32.5)	65 (34.8)	
	≥80	156 (25.7)	109 (25.9)	47 (25.1)	
BMI	<18.5	24 (3.9)	11 (2.6)	13 (7.0)	0.087
	18.6–23.9	322 (53.0)	228 (54.2)	94 (50.3)	
	24–27.9	237 (39.0)	165 (39.2)	72 (38.5)	
	≥28	25 (4.1)	17 (4.0)	8 (4.3)	
Marital status	Single	18 (3.0)	10 (2.4)	8 (4.3)	0.422
	Married	409 (67.3)	287 (68.2)	122 (65.2)	
	Divorced	7 (1.2)	6 (1.4)	1 (0.5)	
	Widowed	174 (28.6)	118 (28.0)	56 (29.9)	
Place of residence	City	385 (63.3)	263 (62.5)	122 (65.2)	0.413
	Town	120 (19.7)	89 (21.1)	31 (16.6)	
	Village	103 (16.9)	69 (16.4)	34 (18.2)	
Living status	Solitude	104 (17.1)	75 (17.8)	29 (15.5)	0.665
	Home	465 (76.5)	321 (76.2)	144 (77.0)	
	Nursing home	39 (6.4)	25 (5.9)	14 (7.5)	
Education	Elementary and below	252 (41.4)	173 (41.1)	79 (42.2)	0.986
	Junior High School	286 (47.0)	199 (47.3)	87 (46.5)	
	High School	62 (10.2)	44 (10.5)	18 (9.6)	
	College	5 (0.8)	3 (0.7)	2 (1.1)	
	Undergraduate and above	3 (0.5)	2 (0.5)	1 (0.5)	
Occupation	Retired	493 (81.1)	343 (81.5)	150 (80.2)	0.453
	Workers	10 (1.6)	8 (1.9)	2 (1.1)	
	Farmer	85 (14.0)	56 (13.3)	29 (15.5)	
	Freelance	2 (0.3)	1 (0.2)	1 (0.5)	
	Employee	6 (1.0)	6 (1.4)	0 (0.0)	
	Unemployed	12 (2.0)	7 (1.7)	5 (2.7)	
Average monthly family income	≤1,000	5 (0.8)	5 (1.2)	0 (0.0)	0.352
	1,000–3,000	289 (47.5)	194 (46.1)	95 (50.8)	
	3,000–5,000	233 (38.3)	166 (39.4)	67 (35.8)	
	>5,000	81 (13.3)	56 (13.3)	25 (13.4)	
Payment method	Medical insurance	604 (99.3)	419 (99.5)	185 (98.9)	0.322
	Self-funded	3 (0.5)	2 (0.5)	1 (0.5)	
	Public expense	1 (0.2)	0 (0.0)	1 (0.5)	
Number of years with VLUs	t ≤ 1	158 (26.0)	106 (25.2)	52 (27.8)	0.340
	1<t ≤ 3	174 (28.6)	128 (30.4)	46 (24.6)	
	t>3	276 (45.4)	187 (44.4)	89 (47.6)	
Number of previous recurrences	0	117 (19.2)	76 (18.1)	41 (21.9)	0.535
	1–3	370 (60.9)	260 (61.8)	110 (58.8)	
	≥4	121 (19.9)	85 (20.2)	36 (19.3)	
Duration of last ulcer (month)	t ≤ 3	165 (27.1)	110 (26.1)	55 (29.4)	0.505
	3<t ≤ 6	191 (31.4)	138 (32.8)	53 (28.3)	
	t>6	252 (41.4)	173 (41.1)	79 (42.2)	
Largest area of last ulcer (cm^2^)	<4	160 (26.3)	107 (25.4)	53 (28.3)	0.647
	4–16	272 (44.7)	197 (46.8)	75 (40.1)	
	16.1–36	108 (17.8)	72 (17.1)	36 (19.3)	
	36.1–80	52 (8.6)	35 (8.3)	17 (9.1)	
	>80	16 (2.6)	10 (2.4)	6 (3.2)	
Venous surgery	Yes	113 (18.6)	79 (18.8)	34 (18.2)	0.865
Previous DVT	Yes	99 (16.3)	70 (16.6)	29 (15.5)	0.730
Heart disease	Yes	221 (36.3)	148 (35.2)	73 (39.0)	0.358
Rheumatoid arthritis	Yes	30 (4.9)	21 (5.0)	9 (4.8)	0.927
Diabetes	Yes	290 (47.7)	206 (48.9)	84 (44.9)	0.361
Hypertension	Yes	349 (57.4)	247 (58.7)	102 (54.5)	0.343
Anemia	Yes	44 (7.2)	34 (8.1)	10 (5.3)	0.231
Cancer	Yes	11 (1.8)	8 (1.9)	3 (1.6)	0.801
Renal dysfunction	Yes	25 (4.1)	16 (3.8)	9 (4.8)	0.562
Smoking status	Never	307 (50.5)	212 (50.4)	95 (50.8)	0.799
	Prior	218 (35.9)	149 (35.4)	69 (36.9)	
	Current	83 (13.7)	60 (14.3)	23 (12.3)	
Alcohol consumption status	Never	318 (52.3)	219 (52.0)	99 (52.9)	0.553
	Prior	240 (39.5)	164 (39.0)	76 (40.6)	
	Current	50 (8.2)	38 (9.0)	12 (6.4)	
Daily leg elevation time (min)	t<30	458 (75.3)	317 (75.3)	141 (75.4)	0.978
	t ≥ 30	150 (24.7)	104 (24.7)	46 (24.6)	
Daily exercise time (h)	t<1	397 (65.3)	278 (66.0)	119 (63.6)	0.567
	t ≥ 1	211 (34.7)	143 (34.0)	68 (36.4)	
Daily stress treatment time (h)	t<12	579 (95.2)	400 (95.0)	179 (95.7)	0.705
	t ≥ 12	29 (4.8)	21 (5.0)	8 (4.3)	
Daily living ability	No dysfunction	167 (27.5)	115 (27.3)	52 (27.8)	0.011
	Varying degrees of dysfunction	182 (29.9)	112 (26.6)	70 (37.4)	
	Apparent dysfunction	259 (42.6)	194 (46.1)	65 (34.8)	
Nutrition	malnutrition	22 (3.6)	15 (3.6)	7 (3.7)	0.519
	Risk of malnutrition	212 (34.9)	153 (36.3)	59 (31.6)	
	Normal nutrition	374 (61.5)	253 (60.1)	121 (64.7)	
Frailty	Frailty	279 (45.9)	193 (45.8)	86 (46.0)	0.973
Depression	Depressed	220 (36.2)	154 (36.6)	66 (35.3)	0.761
Self-efficacy	Lower	450 (74.0)	317 (75.3)	133 (71.1)	0.471
	Middle	150 (24.7)	98 (23.3)	52 (27.8)	
	Higher	8 (1.3)	6 (1.4)	2 (1.1)	
Social support	Lower	150 (24.7)	101 (24.0)	49 (26.2)	0.559
	Middle	458 (75.3)	320 (76.0)	138 (73.8)	

BMI, body mass index; DVT, deep vein thrombosis.

### Identify candidate variables

**Figure 1:**
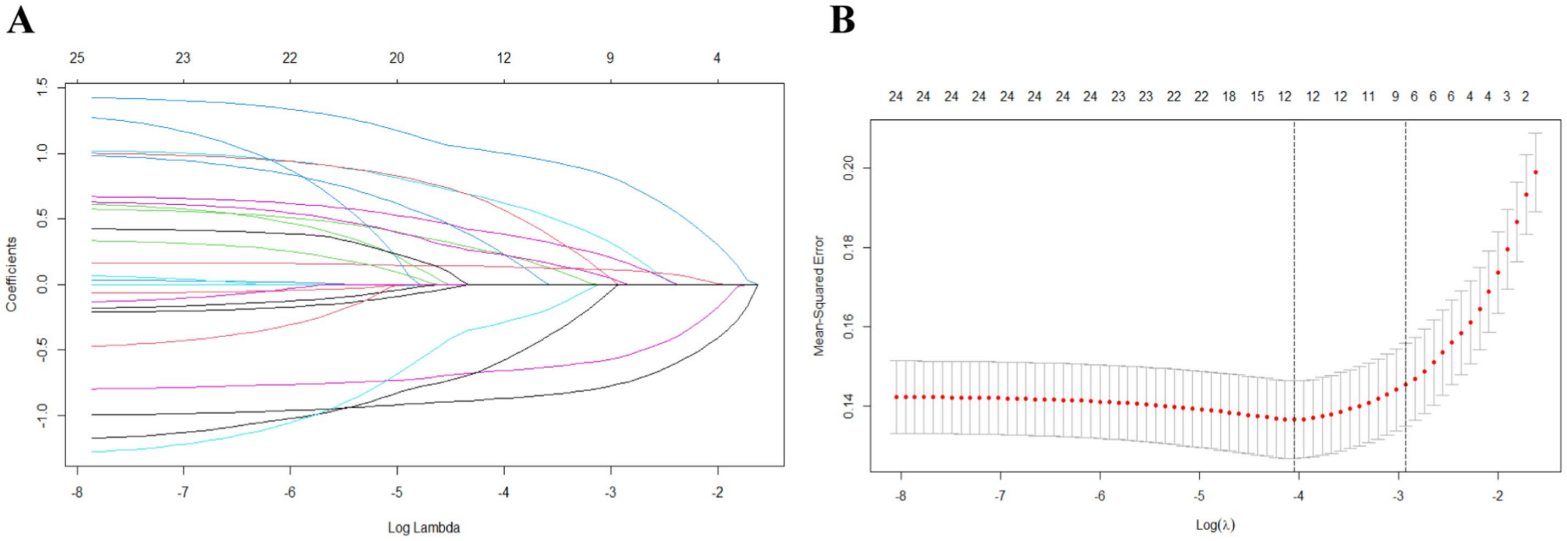
Feature selection using the least absolute shrinkage and selection operator (LASSO) Cox regression model. (A) LASSO coefficient profiles of the 24 features. (A) Coefficient profile plot was produced against the log (*λ*) sequence. (B) Selection of tuning parameter (λ) in the LASSO regression using 10-fold cross-validation via minimum criteria. At the optimal values log (λ), where features are selected, two dotted vertical lines were drawn at the optimal scores by minimum criteria and 1-s.e. criteria.

In this study, candidate variables were selected based on the results of univariate analysis, as well as considering expertise, stability, and accessibility of the variables. The significant variables from the univariate analysis are presented in Table 2, resulting in a total of 17 variables being included as candidates. Furthermore, gender, BMI, education, and maximum size of the last ulcer were added based on expertise, stability, and accessibility, bringing the final count of candidate variables to 21. Then, features with nonzero coefficients were screened out by running the LASSO method. Among the 21 associated variables, nine potential predictor variables (*λ* = 0.0534) were finally retained in the training set based on the non-zero coefficient characteristic variable screening process of LASSO regression (Figures 1A,B). These features were: number of previous recurrences, duration of last ulcer, the history of lower extremity DVT, daily exercise time, daily stress treatment time, ability to perform activities of daily living, frailty, depression, and self-efficacy (Table 3).

**Table 2:** Univariate analyses between patients with and without Recurrence in the training set.

	Characteristic	Non-recurrence group (*n* = 115)	Recurrence group (*n* = 306)	*p-*value
Gender	Male	78 (67.8)	185 (60.5)	0.164
	Female	37 (32.2)	121 (39.5)	
Age	60–69	64 (55.7)	111 (36.3)	0.001^*^
	70–79	33 (28.7)	104 (34.0)	
	≥80	18 (15.7)	91 (29.7)	
BMI	<18.5	5 (4.3)	6 (20.0)	0.498
	18.6–23.9	58 (50.4)	170 (55.6)	
	24–27.9	47 (40.9)	118 (38.6)	
	≥28	5 (4.3)	12 (3.9)	
Marital status	Single	2 (1.7)	8 (2.6)	0.001^*^
	Married	95 (82.6)	192 (62.7)	
	Divorced	2 (1.7)	4 (1.3)	
	Widowed	16 (13.9)	102 (33.3)	
Place of residence	City	69 (60.0)	194 (63.4)	0.813
	Town	26 (22.6)	63 (20.6)	
	Village	20 (17.4)	49 (16.0)	
Living status	Solitude	17 (14.8)	58 (19.0)	0.037^*^
	Home	96 (83.5)	225 (73.5)	
	Nursing home	2 (1.7)	23 (7.5)	
Education	Elementary and below	38 (33.0)	135 (44.1)	0.153
	Junior High School	59 (51.3)	140 (45.8)	
	High School	17 (14.8)	27 (8.8)	
	College	1 (0.9)	2 (0.7)	
	Undergraduate and above	0 (0.0)	2 (0.7)	
Occupation	Retired	96 (83.5)	247 (80.7)	0.308
	Workers	2 (1.7)	6 (20.0)	
	Farmer	11 (9.6)	45 (14.7)	
	Freelance	1 (0.9)	0 (0.0)	
	Employee	3 (2.6)	3 (10.0)	
	Unemployed	2 (1.7)	5 (1.6)	
Average monthly family income	≤1,000	3 (2.6)	2 (0.7)	0.153
	1,000–3,000	46 (40.0)	148 (48.4)	
	3,000–5,000	47 (40.9)	119 (38.9)	
	>5,000	19 (16.5)	37 (12.1)	
Payment method	Medical insurance	115 (100.0)	304 (99.3)	0.385
	Self-funded	0 (0.0)	2 (0.7)	
	public expense	0 (0.0)	0 (0.0)	
Number of years with VLUs	t ≤ 1	34 (29.6)	72 (23.5)	0.091
	1<t ≤ 3	26 (22.6)	102 (33.3)	
	t>3	55 (47.8)	132 (43.1)	
Number of previous recurrences	0	36 (31.3)	40 (13.1)	<0.001^*^
	1–3	67 (58.3)	193 (63.1)	
	≥4	12 (10.4)	73 (23.9)	
Duration of last ulcer (month)	t ≤ 3	55 (47.8)	55 (18.0)	<0.001^*^
	3<t ≤ 6	29 (25.2)	109 (35.6)	
	t>6	31 (27.0)	142 (46.4)	
Largest area of last ulcer (cm^2^)	<4	37 (32.2)	70 (22.9)	0.119
	4–16	50 (43.5)	147 (48.0)	
	16.1–36	20 (17.4)	52 (17.0)	
	36.1–80	8 (7.0)	27 (8.8)	
	>80	0 (0.0)	10 (3.3)	
Venous surgery	Yes	18 (15.7)	61 (19.9)	0.316
Previous DVT	Yes	8 (7.0)	62 (20.3)	0.001^*^
Heart disease	Yes	19 (16.5)	129 (42.2)	<0.001^*^
Rheumatoid arthritis	Yes	9 (7.8)	12 (3.9)	0.101
Diabetes	Yes	56 (48.7)	150 (49.0)	0.953
Hypertension	Yes	60 (52.2)	187 (61.1)	0.097
Anemia	Yes	4 (3.5)	30 (9.8)	0.034^*^
Cancer	Yes	4 (3.5)	4 (1.3)	0.146
Renal dysfunction	Yes	3 (2.6)	13 (4.2)	0.433
Smoking status	Never	58 (50.4)	154 (50.3)	0.664
	Prior	38 (33.0)	111 (36.3)	
	Current	19 (16.5)	41 (13.4)	
alcohol consumption status	Never	60 (52.2)	159 (52.0)	0.799
	Prior	43 (37.4)	121 (39.5)	
	Current	12 (10.4)	26 (8.5)	
Daily leg elevation time (min)	t<30	77 (67.0)	240 (78.4)	0.015^*^
	t ≥ 30	38 (33.0)	66 (21.6)	
Daily exercise time (h)	t<1	40 (34.8)	238 (77.8)	<0.001^*^
	t ≥ 1	75 (65.2)	68 (22.2)	
Daily stress treatment time (h)	t<12	101 (87.8)	299 (97.7)	0.001^*^
	t ≥ 12	14 (12.2)	7 (2.3)	
Daily living ability	No dysfunction	63 (54.8)	52 (17.0)	<0.001^*^
	Varying degrees of dysfunction	31 (27.0)	81 (26.5)	
	Apparent dysfunction	21 (18.3)	173 (56.5)	
Nutrition	malnutrition	3 (2.6)	12 (3.9)	<0.001^*^
	Risk of malnutrition	22 (19.1)	131 (42.8)	
	Normal nutrition	90 (78.3)	163 (53.3)	
Frailty	Frailty	13 (11.3)	180 (58.8)	<0.001^*^
Depression	Depressed	12 (10.4)	142 (46.4)	<0.001^*^
Self-efficacy	Lower	49 (42.6)	268 (87.6)	<0.001^*^
	Middle	63 (54.8)	35 (11.4)	
	Higher	3 (2.6)	3 (10.0)	
Social support	Lower	9 (7.8)	92 (30.1)	<0.001^*^
	Middle	106 (92.2)	214 (69.9)	

BMI, body mass index; DVT, deep vein thrombosis; *p < 0.05.

**Table 3:** Least absolute shrinkage and selection operator regression coefficient table.

Variable	Coefficients	Lambda.1se
Number of previous recurrences	2.869	0.0534
Duration of last ulcer (month)	1.863	
Previous DVT	2.662	
Daily exercise time (h)	−5.626	
Daily stress treatment time (h)	−2.834	
Daily living ability	1.079	
Frailty	7.990	
Depression	2.537	
Self-efficacy	−7.635	

DVT, deep vein thrombosis.

### Construction of the nomogram

**Figure 2:**
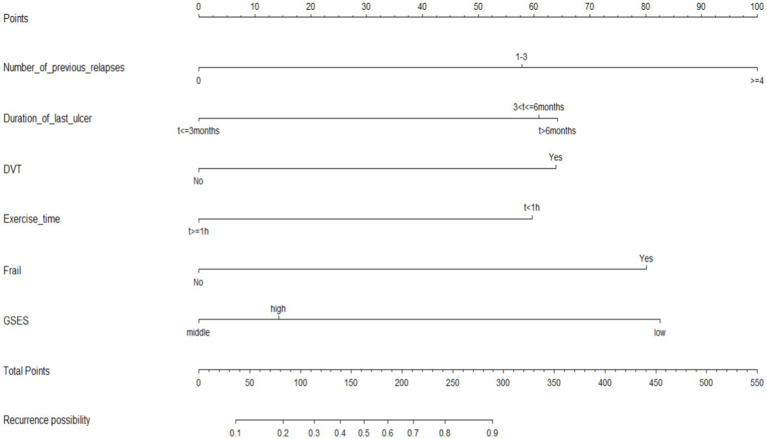
Nomogram model for predicting 6-month wound recurrence in elderly patients with venous leg ulcers.

The nine variables chosen in the LASSO regression analysis were further brought into multivariate logistic regression. The *p*-values of these nine variables, as well as the calculated relative risks, were listed in Table 4. Finally, the prediction model was presented in a Nomogram (Figure 2) and consists of six predictors. These predictors include previous recurrence times (OR = 2.42, 95%CI = 1.50–3.89, *p* < 0.001), last ulcer duration (OR = 1.71, 95%CI = 1.21–2.41, *p* = 0.002), history of deep venous thrombosis of lower extremity (OR = 2.84, 95%CI = 1.15–6.99, *p* = 0.023), existential frailty (OR = 3.70, 95%CI = 1.72–7.95, *p* = 0.001), daily exercise time (OR = 0.46, 95%CI = 0.25–0.86, *p* = 0.016), and self-efficacy (OR = 0.35, 95%CI = 0.19–0.62, *p* < 0.001).

**Table 4:** Multivariate logistic regression analysis of the selected variables in the training set.

Variable	Coefficients	SE	OR	95% CI	*Z*	*p*
Number of previous recurrences	0.882	0.243	2.42	1.50–3.89	3.624	<0.001^*^
Duration of last ulcer (month)	0.538	0.175	1.71	1.21–2.41	3.080	0.002^*^
Previous DVT	1.043	0.460	2.84	1.15–6.99	2.269	0.023^*^
Daily exercise time (h)	−0.768	0.317	0.46	0.25–0.86	−2.420	0.016^*^
Daily stress treatment time (h)	−1.034	0.618	0.36	0.11–1.19	−1.673	0.094
Daily living ability	0.192	0.214	1.21	0.8–1.84	0.899	0.369
Frailty	1.307	0.391	3.70	1.72–7.95	3.346	0.001^*^
Depression	0.450	0.399	1.57	0.72–3.43	1.127	0.260
Self-efficacy	−1.064	0.298	0.35	0.19–0.62	−3.569	<0.001^*^

DVT, deep vein thrombosis; The C-statistic value: 0.869; *p < 0.05.

### Validation of the nomogram

**Figure 3:**
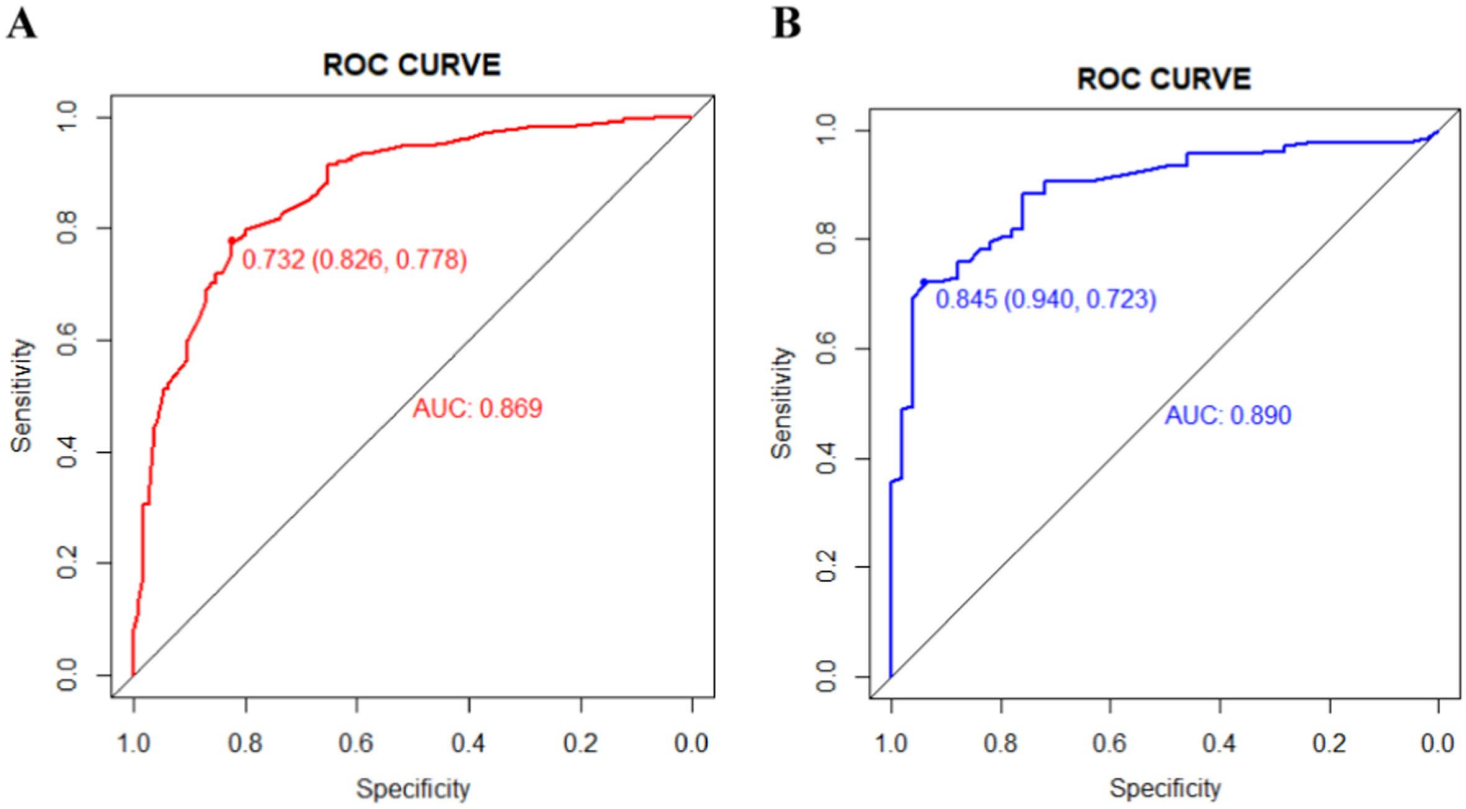
The area under the receiver operating characteristic (ROC) curves (AUCs) of the nomogram for predicting wound recurrence in training set (A) and validation set (B).

**Figure 4:**
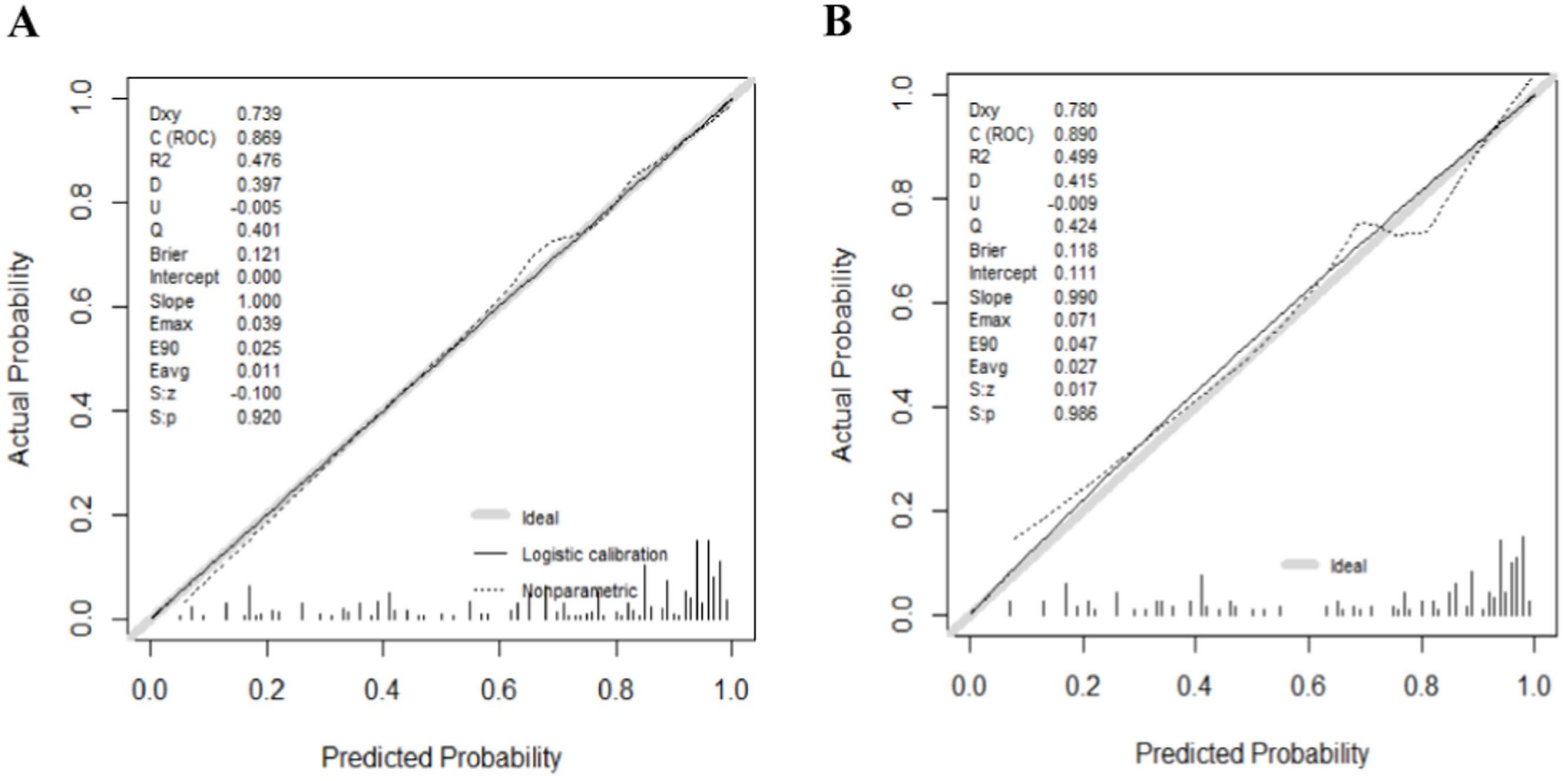
The logistic calibration curve of the nomogram for predicting wound recurrence in training set (A) and validation set (B).

The ROC curves were generated for the training and validation sets, with AUC values of 0.869 (95%CI: 0.831–0.908) in the training set (Figure 3A) and 0.890 (95%CI: 0.841–0.938) in the validation set (Figure 3B).The C-index of the nomogram in both sets was greater than 0.70, indicating a good discriminative ability of the model. Calibration curves of the nomogram in the training and validation sets demonstrated a favorable consistency between the predicted and actual probabilities (Figures 4A,B). The Hosmer-Lemeshow test results for the nomogram in the training and validation sets were *χ*^2^ = 4.35 (*p* = 0.887) and *χ*^2^ = 6.17 (*p* = 0.772) respectively. The insignificant *p*-values of the HL test indicated a good fit of the nomogram in both sets.

## Discussion

In this study, a total of 608 elderly patients with VLUs were included. Out of these patients, 443 experienced ulcer recurrence within 6 months after wound healing, resulting in a 6-month ulcer recurrence rate of 72.9% for elderly VLUs patients. This finding is slightly higher than the epidemiologic recurrence rate of 50 to 70 percent for VLUs patients at 6 months (1). Reeder et al. (21) conducted a follow-up study on the recurrence rate of ulcers in patients with refractory VLUs after discharge. Their results showed that 85.3% of patients experienced recurrence within 2 months, which may be attributed to the fact that the subjects of their study had refractory VLUs. Tzaneza et al. (22) conducted a single-center retrospective cohort study and found that the recurrence rate of ulcers within 12 months was 60%, with most recurrences occurring in the first 3 months. The higher recurrence rate observed in our study compared to other relevant studies could be due to the inclusion of elderly patients who have a higher prevalence of VLUs and are less capable of self-care, making them more prone to relapse (23, 24).

The relationship between the number of previous recurrences and the risk of wound recurrence in VLUs patients has been discussed in relevant studies. In patients with 1 to 3 previous recurrences, the risk of wound recurrence after 6 months was 2.42 times higher (95%CI: 1.50–3.89). Furthermore, the risk of recurrence increases with the number of previous recurrences. The study found that 94% of patients with recurrence had experienced multiple ulcers in the past, and the probability of recurrence was 4.4 times higher for patients with two or more previous ulcers compared to those with an initial recurrence (15).

The previous study findings indicate a positive correlation between the duration of the last ulcer and wound recurrence in patients with VLUs (25, 26). Finlayson et al. (15) observed that the risk of recurrence increased by 1.005 times for each additional week of ulcer duration. Similarly, Tzaneva et al. (22) demonstrated that the recurrence risk increased by 1.020 times for every additional month of ulcer duration. Another study reported that after VLUs persisted for 1 month, the probability of recurrence after 1 year was 25.1%, and this probability increased to 74.9% after VLUs persisted for 16 months (27). According to our model, patients with a last ulcer duration longer than 6 months had a 1.71-fold greater risk of recurrence compared to those with a duration of 3–6 months (95% CI: 1.21–2.41). Consequently, timely treatment of VLUs wounds is crucial to promote prompt healing.

The history of lower extremity DVT is widely recognized as a significant risk factor for VLUs recurrence. In this study, it was found that patients with a prior DVT had 2.84 times the risk of ulcer recurrence within 6 months compared to patients without a history of DVT (95%CI: 1.15–6.99). Previous research also identified a previous history of lower extremity DVT as a risk factor for recurrence, with the recurrence probability being 1.7 times higher in patients with previous DVT compared to those without DVT (13, 15). Lower extremity DVT syndrome, characterized by blocked venous return and venous insufficiency, significantly increases the risk of VLUs, making it more challenging to treat and more prone to recurrence (28).

Previous studies have confirmed that exercise is a protective factor for wound recurrence in patients with VLUs. In this study, the risk of recurrence was found to be 0.46 times higher in patients with a daily exercise duration of less than 1 h compared to those with a daily exercise duration of 1 h or more (95% CI: 0.25–0.86). Finlayson et al. (15) also confirmed in their study that participating in appropriate exercise programs, such as walking, can effectively reduce the recurrence rate of VLUs patients. Patients who walk for at least 3 h a day have a recurrence rate 1.6 times lower than those who exercise for less than 3 h a day. Meagher et al. (29) further found that encouraging VLUs patients to increase their daily step count to 10,000 steps resulted in faster healing compared to those who did not change their step count. This indirectly suggests that walking can improve the pump function of the calf muscle, leading to a reduction in recurrence.

This study utilized the Frail scale to assess the presence of frailty in patients and found that those with frailty had a 3.70 times higher risk of VLUs recurrence after 6 months compared to patients without frailty (95% CI: 1.72–7.95). This study demonstrates that frailty is an independent predictor of wound recurrence in elderly patients with VLUs. This is explained by the fact that the population of this study was elderly and frailty is a geriatric syndrome associated with aging that often leads to negative health outcomes (30).

The role of self-efficacy in preventing wound recurrence in elderly VLUs patients should also be considered. According to this model, a higher sense of self-efficacy is associated with a lower risk of relapse. Finlayson et al. (15) have also found a negative correlation between self-efficacy and relapse. The level of self-efficacy directly influences patients’ self-management, with higher self-efficacy leading to more active wound management (31). High levels of self-efficacy are closely linked to patients’ self-management and health outcomes (32, 33). Patients with high self-efficacy demonstrate greater motivation, better self-management skills, and higher compliance, which ultimately contributes to better health outcomes. These findings highlight the importance of implementing self-management programs aimed at enhancing self-efficacy.

This study presents a prediction model for wound recurrence in elderly patients with VLUs. The model was employed a combination of single-factor analysis and the Lasso algorithm for variable screening. The Lasso algorithm utilizes a penalty term to reduce the parameter value of variables with low weight to zero, thereby enhancing the stability of the model at the cost of potential estimation deviation. The constructed prediction model demonstrated AUC values of 0.869 (95%CI: 0.831–0.908) in the training set and 0.890 (95%CI: 0.841–0.938) in the validation set. A higher AUC value closer to 1 indicates a greater degree of differentiation and accuracy above 0.9. Thus, this model exhibits good prediction ability and can accurately calculate the probability of wound recurrence in elderly VLUs patients. The H-L test *p*-values of the prediction model in the training set and validation set were 0.887 and 0.772, respectively, both of which were greater than 0.05. Calibration degree plots revealed a close alignment of data points with the diagonal line, indicating that the predicted probability of wound recurrence in elderly VLUs patients was in close agreement with the actual probability. Therefore, the prediction model demonstrates good prediction ability. This study is a multi-center investigation, with the sample size calculated using a specific formula. The final dataset comprises 608 patients sourced from five medical institutions, including tertiary general hospitals, secondary medical facilities, primary care settings, and wound care outpatient clinics. This diverse representation enhances the study’s generalizability, establishing it as a pilot study. However, it is important to note that this study only conducted internal validation of the prediction model and did not perform external validation. Consequently, the generalizability of the model to a broader population remains uncertain, necessitating external validation. Moving forward, we plan to validate the model using an external cohort to enhance the accuracy of our findings. Additionally, this prediction model will be further extended and analyzed. For instance, based on this risk prediction model, the future development of simple and diverse small programs or software can be envisaged, which may provide personalized guidance to patients through corresponding prevention or intervention strategies.

Currently, most patients with venous leg ulcers who experience relapse are evaluated by medical staff based on clinical characteristics and their professional experience. By integrating the risk prediction model developed in this study into clinical practice, it becomes possible to identify the recurrence of VLUs in elderly patients through early screening. This proactive approach allows for the formulation of intervention plans in advance, such as developing exercise intervention strategies tailored for VLU patients, implementing effective measures to enhance self-efficacy, prevent frailty, and promote a more precise, individualized, and scientific approach to clinical care. Additionally, patients can be guided to self-manage controllable risk factors associated with VLUs, including daily exercise duration, fitness levels, self-efficacy, and other relevant factors, while also encouraging early treatment of any wounds to minimize ulcer duration. Ultimately, the goal is to reduce the risk of recurrence in elderly VLU patients, enhance their quality of life, and optimize the utilization of medical costs and resources.

## Conclusion

In this study, a nomogram was developed to predict wound recurrence in elderly patients with VLUs. The nomogram consists of six predictors that accurately estimate the probability of wound recurrence within 6 months. This nomogram holds significance in forecasting wound recurrence in elderly VLUs patients and can assist healthcare professionals in determining the need for early intervention based on prediction outcomes and individual patient circumstances.

## Data Availability

The original contributions presented in the study are included in the article/supplementary material, further inquiries can be directed to the corresponding author.

## References

[1] O’Donnell TF Jr Passman MA Marston WA Ennis WJ Dalsing M Kistner RL. Management of venous leg ulcers: clinical practice guidelines of the Society for Vascular Surgery ® and the American venous forum. J Vasc Surg. (2014) 60:3s–59s. doi: 10.1016/j.jvs.2014.04.049, PMID: 24974070

[2] Weller CD Richards C Turnour L Team V. Venous leg ulcer management in Australian primary care: patient and clinician perspectives. Int J Nurs Stud. (2021) 113:103774. doi: 10.1016/j.ijnurstu.2020.103774, PMID: 33080480

[3] Ratliff CR Yates S McNichol L Gray M. Compression for primary prevention, treatment, and prevention of recurrence of venous leg ulcers: an evidence-and consensus-based algorithm for Care across the continuum. J Wound Ostomy Continence Nurs. (2016) 43:347–64. doi: 10.1097/WON.0000000000000242, PMID: 27163774 PMC4937809

[4] Franks PJ Barker J Collier M Gethin G Haesler E Jawien A. Management of Patients with Venous leg Ulcers: challenges and current best practice. J Wound Care. (2016) 25:S1–s67. doi: 10.12968/jowc.2016.25.Sup6.S1, PMID: 27292202

[5] Abbade LP Lastória S. Venous ulcer: epidemiology, physiopathology, diagnosis and treatment. Int J Dermatol. (2005) 44:449–56. doi: 10.1111/j.1365-4632.2004.02456.x, PMID: 15941430

[6] Taylor RR Sladkevicius E Guest JF. Modelling the cost-effectiveness of electric stimulation therapy in non-healing venous leg ulcers. J Wound Care. (2011) 20:464–72. doi: 10.12968/jowc.2011.20.10.464, PMID: 22067884

[7] Weller CD Buchbinder R Johnston RV Cochrane Wounds Group. Interventions for helping people adhere to compression treatments for venous leg ulceration. Cochrane Database Syst Rev. (2016) 2016:CD008378. doi: 10.1002/14651858.CD008378.pub3, PMID: 26932818 PMC6823259

[8] Kalra M Gloviczki P. Surgical treatment of venous ulcers: role of subfascial endoscopic perforator vein ligation. Surg Clin North Am. (2003) 83:671–705. doi: 10.1016/S0039-6109(02)00198-6, PMID: 12822732

[9] Chase SK Melloni M Savage A. A forever healing: the lived experience of venous ulcer disease. J Vasc Nurs. (1997) 15:73–8. doi: 10.1016/S1062-0303(97)90004-2, PMID: 9238945

[10] Hareendran A Bradbury A Budd J Geroulakos G Hobbs R Kenkre J. Measuring the impact of venous leg ulcers on quality of life. J Wound Care. (2005) 14:53–7. doi: 10.12968/jowc.2005.14.2.26732, PMID: 15739651

[11] Layer A McManus E Levell NJ. A systematic review of model-based economic evaluations of treatments for venous leg ulcers. PharmacoEconomics. (2020) 4:211–22. doi: 10.1007/s41669-019-0148-x, PMID: 31134471 PMC7248160

[12] Brown A. Does social support impact on venous ulcer healing or recurrence? Br J Community Nurs. (2008) 13:S8–S15. doi: 10.12968/bjcn.2008.13.Sup1.28687, PMID: 18557569

[13] Finlayson K Edwards H Courtney M. Relationships between preventive activities, psychosocial factors and recurrence of venous leg ulcers: a prospective study. J Adv Nurs. (2011) 67:2180–90. doi: 10.1111/j.1365-2648.2011.05653.x, PMID: 21517938

[14] Brooks J Ersser SJ Lloyd A Ryan TJ. Nurse-led education sets out to improve patient concordance and prevent recurrence of leg ulcers. J Wound Care. (2004) 13:111–6. doi: 10.12968/jowc.2004.13.3.26585, PMID: 15045806

[15] Finlayson K Wu ML Edwards HE. Identifying risk factors and protective factors for venous leg ulcer recurrence using a theoretical approach: a longitudinal study. Int J Nurs Stud. (2015) 52:1042–51. doi: 10.1016/j.ijnurstu.2015.02.016, PMID: 25801312

[16] Lawton MP Brody EM. Assessment of older people: self-maintaining and instrumental activities of daily living. Gerontologist. (1969) 9:179–86. doi: 10.1093/geront/9.3_Part_1.179, PMID: 5349366

[17] Rubenstein LZ Harker JO Salvà A Guigoz Y Vellas B. Screening for undernutrition in geriatric practice: developing the short-form mini-nutritional assessment (MNA-SF). J Gerontol A Biol Sci Med Sci. (2001) 56:M366–72. doi: 10.1093/gerona/56.6.M366, PMID: 11382797

[18] Cacciatore F Testa G Galizia G Della-Morte D Mazzella F Langellotto A. Clinical frailty and long-term mortality in elderly subjects with diabetes. Acta Diabetol. (2013) 50:251–60. doi: 10.1007/s00592-012-0413-2, PMID: 22732903

[19] Yesavage JA Sheikh JI. 9/geriatric depression scale (GDS). Clin Gerontol. (1986) 5:165–73. English. doi: 10.1300/J018v05n01_09

[20] Okimoto JT Barnes RF Veith RC Raskind MA Inui TS Carter WB. Screening for depression in geriatric medical patients. Am J Psychiatry. (1982) 139:799–802. doi: 10.1176/ajp.139.6.799, PMID: 7081496

[21] Reeder S de Roos KP de Maeseneer M Sommer A Neumann HA. Ulcer recurrence after in-hospital treatment for recalcitrant venous leg ulceration. Br J Dermatol. (2013) 168:999–1002. doi: 10.1111/bjd.12164, PMID: 23253015

[22] Tzaneva S Heere-Ress E Kittler H Böhler K. Surgical treatment of large vascular leg ulcers. Dermatologic Surg. (2014) 40:1240–8. doi: 10.1097/DSS.0000000000000137, PMID: 25310752

[23] Probst S Seppänen S Gerber V Hopkins A Rimdeika R Gethin G. EWMA document: home care-wound care: overview, challenges and perspectives. J Wound Care. (2014) 23:S1–s41. doi: 10.12968/jowc.2014.23.Sup5a.S1, PMID: 25192441

[24] Kelechi TJ Johnson JJ WOCN Society. Guideline for the management of wounds in patients with lower-extremity venous disease: an executive summary. J Wound Ostomy Continence Nurs. (2012) 39:598–606. doi: 10.1097/WON.0b013e31827179e9, PMID: 23138493

[25] Gohel MS Taylor M Earnshaw JJ Heather BP Poskitt KR Whyman MR. Risk factors for delayed healing and recurrence of chronic venous leg ulcers--an analysis of 1324 legs. Eur J Vasc Endovasc Surg. (2005) 29:74–7. doi: 10.1016/j.ejvs.2004.10.002, PMID: 15570275

[26] Barwell JR Ghauri ASK Taylor M Deacon J Wakely C Poskitt KR. Risk factors for healing and recurrence of chronic venous leg ulcers. Phlebology. (2000) 15:49–52. doi: 10.1177/026835550001500202, PMID: 39061296

[27] Ashby RL Gabe R Ali S Saramago P Chuang LH Adderley U. VenUS IV (venous leg ulcer study IV) - compression hosiery compared with compression bandaging in the treatment of venous leg ulcers: a randomised controlled trial, mixed-treatment comparison and decision-analytic model. Health Technol Assess. (2014) 18:1–294. doi: 10.3310/hta18570, PMID: 25242076 PMC4781202

[28] Labropoulos N Waggoner T Sammis W Samali S Pappas PJ. The effect of venous thrombus location and extent on the development of post-thrombotic signs and symptoms. J Vasc Surg. (2008) 48:407–12. doi: 10.1016/j.jvs.2008.03.016, PMID: 18515036

[29] Meagher H Ryan D Clarke-Moloney M O'Laighin G Grace PA. An experimental study of prescribed walking in the management of venous leg ulcers. J Wound Care. (2012) 21:421–30. doi: 10.12968/jowc.2012.21.9.421, PMID: 22990394

[30] Khan N Hewson D Randhawa G. Effectiveness of integrated chronic care interventions for older people with different frailty levels: a systematic review protocol. BMJ Open. (2020) 10:e038437. doi: 10.1136/bmjopen-2020-038437, PMID: 32912991 PMC7485241

[31] Kapp S Santamaria N. How and why patients self-treat chronic wounds. Int Wound J. (2017) 14:1269–75. doi: 10.1111/iwj.12796, PMID: 28782223 PMC7950121

[32] Dutton GR Tan F Provost BC Sorenson JL Allen B Smith D. Relationship between self-efficacy and physical activity among patients with type 2 diabetes. J Behav Med. (2009) 32:270–7. doi: 10.1007/s10865-009-9200-0, PMID: 19156510

[33] Curtin RB Walters BA Schatell D Pennell P Wise M Klicko K. Self-efficacy and self-management behaviors in patients with chronic kidney disease. Adv Chronic Kidney Dis. (2008) 15:191–205. doi: 10.1053/j.ackd.2008.01.006, PMID: 18334246

